# ENDOLUNG trial. A phase 1/2 study of the Akt/mTOR inhibitor and autophagy inducer Ibrilatazar (ABTL0812) in combination with paclitaxel/carboplatin in patients with advanced/recurrent endometrial cancer

**DOI:** 10.1186/s12885-024-12501-5

**Published:** 2024-07-22

**Authors:** Alexandra Leary, Purificación Estévez-García, Renaud Sabatier, Isabelle Ray-Coquard, Margarita Romeo, Pilar Barretina-Ginesta, Marta Gil-Martin, Elena Garralda, Joaquim Bosch-Barrera, Teresa Morán, Paloma Martin-Martorell, Ernest Nadal, Pere Gascón, Jordi Rodon, Jose M Lizcano, Pau Muñoz-Guardiola, Gemma Fierro-Durán, Oriol Pedrós-Gámez, Héctor Pérez-Montoyo, Marc Yeste-Velasco, Marc Cortal, Antonio Pérez-Campos, Jose Alfon, Carles Domenech, Alejandro Pérez-Fidalgo, Ana Oaknin

**Affiliations:** 1 Medical Oncology, Institut Gustave Roussy, Villejuif, France; 2 Medical Oncology, Hospital Universitario Virgen del Rocío, Sevilla, Spain; 3 Department of Medical Oncology, Aix-Marseille Univ, Inserm, CNRS, Institut Paoli-Calmettes, 232 Boulevard Sainte Marguerite, Marseille, France; 4 Medical Oncology, Centre Léon Bérard, Lyon, France; 5 Department of Medical Oncology, Department of Medicine, Institut Català d’Oncologia Badalona Hospital Germans Trias i Pujol, Badalona-Applied Research Group in Oncology, Germans Trias i Pujol Institute, Universitat Autònoma de Barcelona, Badalona, Spain; 6 Medical Oncology Department, Precision Oncology Group (OncoGIR-Pro) Girona Biomedical Research Institute (IDIBGI) and Department of Medical Sciences, Institut Català d’Oncologia (ICO), Medical School University of Girona (UdG), Girona, Spain; 7 Medical Oncology, Catalan Institute of Oncology and IDIBELL, L’Hospitalet del Llobregat, Barcelona, Spain; 8 Medical Oncology Service, Vall d’Hebron Institute of Oncology (VHIO), Vall d’Hebron Barcelona Hospital Campus, Barcelona, Spain; 9 Medical Oncology, INCLIVA-Hospital Clínico Universitario, Valencia, Spain; 10 Medical Oncology Service, Hospital Clínic. Universitat de Barcelona, Barcelona, Spain; 11 Early Drug Development, The University of Texas M. D. Anderson Cancer Center, Houston, TX USA; 12 Department of Biochemistry and Molecular Biology, Institut de Neurocièncias, Universitat Autònoma de Barcelona, Cerdanyola del Vallès, Barcelona, Spain; 13 Protein Kinases in Cancer Research, Vall Hebron Institut de Recerca (VHIR), Barcelona, Spain; 14 Research and Development, Ability Pharmaceuticals, Cerdanyola del Vallès, Barcelona, Spain

**Keywords:** Endometrial cancer, Autophagy, Chemotherapy, Phase 1/2, Safety profile

## Abstract

**Background:**

Carboplatin and paclitaxel (CP) have been the standard of care for advanced/recurrent endometrial cancer (EC) for many years. However, this chemotherapy combination shows limited efficacy and recurrences often occur in less than 12 months. ABTL0812 is a novel drug that selectively kill cancer cells by cytotoxic autophagy and has shown anticancer efficacy in preclinical models of EC in combination with CP.

**Methods:**

ENDOLUNG was an open-label, phase 1/2 clinical trial designed to determine the safety and efficacy of Ibrilatazar (ABTL0812) with CP in patients with advanced/recurrent EC and non-irradiable stage III and IV squamous non-small cell lung cancer (sq-NSCLC). The phase 1 part consisted of a 3 + 3 de-escalation design followed by an expansion cohort with 12 patients. The primary endpoint was safety. ABTL0812 starting dose was 1300 mg tid combined with carboplatin at area under the curve (AUC) 5 and paclitaxel at 175 mg/m^2^ both administered every 21 days for up to 8 cycles. The phase 2 part included a total of 51 patients. The primary endpoint was overall response rate (ORR) and the secondary endpoints included duration of response (DOR), progression-free survival (PFS) and overall survival (OS).

**Results:**

During the phase 1 only one dose limiting toxicity (DLT), a grade 4 neutropenia, was observed in 1 out of 6 patients, thus no de-escalation was applied. One additional DLT, a grade 3 febrile neutropenia, was observed in the expansion cohort, thus the recommended phase 2 dose (RP2D) for ABTL0812 was established at 1300 mg tid. Most frequent hematological adverse events (AE) of the combination were neutropenia (52.9%), anemia (37.3%) and thrombocytopenia (19.6%). Nausea (66.7%), asthenia (66.7%), diarrhea (54.9%) and vomiting (54.9%) were the most frequent non-hematological adverse events (AEs). The combination of ABTL0812 plus CP showed an ORR of 65.8% (13.2% complete response and 52.6% partial response) with a median DOR of 7.4 months (95% CI: 6.3–10.8 months). Median PFS was 9.8 months (95% CI: 6.6–10.6) and median OS 23.6 months (95% CI 6.4-ND). Pharmacokinetic parameters were compatible with target engagement observed in preclinical studies, and blood pharmacodynamic biomarkers indicated sustained target regulation during, at least, 28 days after starting the treatment.

**Conclusions:**

This study suggests that the combination of ABTL0812 with CP is safe and feasible with an encouraging activity in patients with advanced/recurrent EC. Our data warrant further confirmation in prospective randomized trials.

**Trial registration:**

EU Clinical Trial Register, EudraCT number 2016-001352-21 and National Clinical Trials Number, NCT03366480. Registration on 19 September 2016.

## Highlights

ABTL0812 is a novel drug in clinical development that kills tumor cells by cytotoxic autophagy.

The recommended phase 1/2 dose of ABTL0812 in combination with paclitaxel/carboplatin is 1300 mg three times a day (tid) by the oral route.

The combination of ABTL0812 plus carboplatin and paclitaxel (CP) in patients with advanced/recurrent EC suggests an improved benefit-to-risk ratio vs. CP alone and warrants further clinical evaluation.

## Background

EC is a frequent tumor in women with a heterogeneous behavior. Although most patients will be diagnosed at an early stage and potentially cured after radical therapy [1], in some cases the disease will recur, while others are diagnosed with a de novo metastatic disease. In these last cases, 5-year OS rate ranges between 20 and 25% [2]. Since the GOG0209 trial, the combination of carboplatin AUC 5–6 plus paclitaxel 175 mg/m^2^ every 21 days for six cycles has been the standard of care as a first line treatment in advanced endometrial cancer. This combination has shown an overall response rate (ORR) of 40–50% and a median progression-free (PFS) and overall survival (OS) of 14 and 32 months, respectively [3].

Hyperactivation of the PI3K/Akt/mTOR (PAM) pathway is a common alteration in EC. The most frequent abnormalities related to this pathway are loss of PTEN, gain-of-function mutations of *PKI3CA*, or AKT amplification [4]. These alterations confer an increased aggressivity and poor outcomes to those patients. Therefore, the blockade of this pathway is an attractive therapeutic strategy in EC and other tumor types with altered PAM pathway [5]. Hyperactivation of the PAM pathway can usually lead to the suppression of autophagy [6]. Autophagy is a highly conserved cellular process that degrades unnecessary or dysfunctional components of the cell through a regulated mechanism for cell homeostasis and adaptation to stress. In human cancers, including EC, autophagy may induce antitumor effects depending on stage and other factors [7, 8]. Sustained activation of autophagy can induce cancer cell death; therefore, activation of cytotoxic autophagy is being investigated as an innovative and promising anti-cancer therapeutic strategy [9, 10].

Ibrilatazar (ABTL0812) is a first-in-class orally administered small molecule that kills cancer cells through the induction of cytotoxic autophagy by a dual mechanism of action that includes the inhibition of the Akt/mTORC1 axis by overexpression of the TRIB3 pseudokinase [11], and induction of endoplasmic reticular (ER) stress and, consequently, of the Unfolded Protein Response (UPR) [12]. Both actions converge to induce a robust and persistent autophagy that results in the selective death of cancer cells while sparing normal cells. Importantly, ABTL0812 has been assessed in preclinical EC models, demonstrating efficacy as a single agent and in combination with standard chemotherapy. Thus, in murine models of EC patient-derived xenografts (PDX) showed anticancer activity without added toxicity and inhibited carcinogenesis [13]. A first-in-human (FIH) study in advanced solid tumors as a single agent showed promising results, with no maximum tolerated dose (MTD) reached [14]. The recommended phase 2 dose was determined through pharmacokinetics (PK)/pharmacodynamic (PD) modeling. Most drug-related adverse events were mild gastrointestinal issues. Notably, two cases of long-term stable disease (> 1 year) were observed [14].

The ENDOLUNG phase 1/2 clinical trial was designed to assess the combination of ABTL0812 with CP in patients with metastatic/recurrent EC and advanced squamous non-small cell lung cancer (sq-NSCLC). In the phase 1 part of the study the RP2D of ABTL0812 was determined in a cohort that included both types of tumors. In the phase 2, safety, efficacy, pharmacokinetics and pharmacodynamic biomarkers of the drug were determined in each indication separately. In this study, we describe the phase 1 part (EC and sq-NSCLC) and the cohort of phase 2 that only included patients with EC. Trial registration NCT03366480 at ClinicalTrials.gov.

## Methods

### Study design and conduct

ENDOLUNG was an open-label phase 1/2 study of the combination of ABTL0812 plus CP in advanced or recurrent EC patients (the phase 1 also included patients with sq-NSCLC). Patients were recruited in 9 academic institutions, located in Spain and France, from November 2016 to February 2020. This study was registered with EudraCT number 2016-001352-21 and at ClinicalTrials.gov with identifier NCT03366480.

During the phase 1, patients were recruited only in 6 academic institutions from Spain. The phase 1 stage had a 3 + 3 de-escalation design followed by an expansion part where the participants were scheduled to receive intravenous carboplatin area under the curve (AUC) 5 and paclitaxel 175 mg/m^2^ every 21 days for up to 8 cycles plus ABTL0812. The starting dose level of ABTL0812 had been identified as 1300 mg three times per day (tid) by PK/PD modelling in the FIH study of ABTL0812 administrating the drug as single agent in patients with advanced solid tumors [14]. The feasible safety profile of ABTL0812 both, as single agent in patients, and in combination with CP in the toxicologic preclinical data, suggested that a de-escalation design would be more appropriate.

ABTL0812 (100 mg/mL oral solution, supplied by Ability Pharmaceuticals SA) de-escalation dose levels where 1000, 650 and 500 mg tid. Intrapatient de-escalation was not permitted. Drug treatment with ABTL0812 started 7 days before the first cycle of chemotherapy with the objective of attaining stable intra-tumor drug levels. ABTL0812 was subsequently administered, daily, until disease progression, unacceptable toxicity, informed consent withdrawal or investigator’s decision.

Dose Limiting Toxicities (DLTs) that appeared in more than 1 in 6 patients treated during the de-escalation part or more than 2 in 12 in the expansion cohort was considered the criteria for dose de-escalation. DLTs were defined as the following AEs appearing from inclusion until the last day of the first chemotherapy cycle: grade 4 neutropenia lasting more than 7 days^1^, grades 3 and 4 febrile neutropenia, grade 3 thrombocytopenia without bleeding lasting for more than 7 consecutive days, grade 3 thrombocytopenia with signs of bleeding or requiring transfusion, grade 4 thrombocytopenia, grade 3 or more nausea and vomiting lasting more than 3 days even when optimal prophylactic or therapeutic measures were administered, grade 3 or more AST and ALT elevation lasting 7 or more days and any other grade 3 or more non-hematological toxicities with the exception of grade 3 GGT elevation or other grade 3 laboratory test without clinical relevance as per physician’s evaluation. A selection of oncologists participating in the study and company representatives constituted a Data Monitoring which had, among others, the responsibility of monitoring AEs, determining if DLTs had appeared during the first cycle of chemotherapy and if they were attributable to the study drug.

For the phase 2 part, a Simon´s two stage design was performed. ABTL0812 was administered at the RPD2 determined from the phase 1 part plus CP at the same doses of the phase 1 part.

### Endpoints and assessments

For the phase 1 part, the primary endpoint was safety and tolerability of the combination according to Common Terminology Criteria for Adverse Events (CTCAE) v4.03. Safety and tolerability were monitored by physical examination, ECG, hematology and clinical biochemistry, urinalysis, and assessment of ECOG performance status. A secondary endpoint of the phase 1 was the determination of the RP2D.

For the phase 2 part, the primary endpoint was ORR. Secondary endpoints were PFS (median and progression-free patients’ rate at 12 months), and DOR. Median PFS was defined as the time from the administration of the first dose of ABTL0812 to recurrence or death (whatever first) and DOR was defined as the time from first response until renewed tumor progression was observed. Efficacy’s evaluation was based on the investigator’s assessment of tumor by CT-scans performed at baseline and then every 8 weeks. Definition of measurable and non-measurable lesions, the determination of their size and the criteria for tumor evaluation was performed according to RECIST v1.1 criteria [15]. OS was defined as time from first dose administration to death from any cause. Long term follow-up was censored at 2 years.

Secondary endpoints of the phase 2 part of the study included pharmacokinetics (PK) of ABTL0812 in plasma and determination of pharmacodynamic (PD) biomarkers of drug activity.

### Patient eligibility

For the phase 1 part both endometrial and lung cancer patients were included. In the EC cohort patients were required to have a histologic diagnosis of advanced (metastatic or recurrent) EC. All histological types were eligible except carcinosarcoma and uterine sarcoma. In the lung cohort, patients should have a histological diagnosis of squamous non-small cell lung cancer (sq-NSCLC). Non irradiable sq-NSCLC stage III or stage IV were included. Other lung cancer subtypes such as mixed tumors, neuroendocrine or adenocarcinoma tumors were excluded.

For all tumor types, patients should accomplish criteria for measurable disease as per Response Evaluation Criteria in Solid Tumors (RECIST) version 1.1 with at least one target lesion to be used to assess the response. Progressive lesions within a previously irradiated field were designated as non-target lesions unless progression was documented. Eastern Cooperative Oncology Group (ECOG) Performance Status should had been 0 or 1. Bone marrow function criteria was defined by absolute neutrophil count ≥ 1.5 × 10^9^/L, platelet count ≥ 100 × 10^9^/L and hemoglobin ≥ 10.0 g/dL. Total bilirubin should had been ≤ 1.5 x upper limit of normal (ULN); AST ≤ 2.5 times ULN (or ≤ 5 times the ULN in patients with evidence of liver metastases); and alkaline phosphatase (ALP) ≤ 2.5 times ULN (or ≤ 5 times the ULN in patients with evidence of liver metastases) and serum creatinine ≤ 1.5 ULN.

Patients were excluded if they had been previously treated with an inhibitor of the PI3K/Akt/mTOR pathway; had adjuvant chemotherapy or radiotherapy administered less than 6 months before inclusion; had symptomatic brain metastases; had gastrointestinal abnormalities including inability to take oral medications, malabsorption syndromes or other clinically significant gastrointestinal abnormalities. Granulocyte colony-stimulating factors were allowed.

### Pharmacokinetic and pharmacodynamic biomarkers

EDTA-anticoagulated blood samples for PK analysis were obtained on the first day of the run-in treatment with ABTL0812 and on the first day of the second cycle of chemotherapy (being an interval period of 28 days between them). Serial samples were immediately centrifuged, plasma separated, frozen and stored − 80 °C. Bioanalysis of ABTL0812 enantiomers was performed using a validated method by Echevarne’s Laboratory (Sant Cugat del Valles, Spain). The pharmacokinetic determination of plasma ABTL0812 enantiomers concentrations was performed at the Faculty of Pharmacy of the University of Barcelona (Spain). The pharmacokinetic metrics were calculated using the non-compartmental approach using the Phoenix-WinNonlin ver.8.6.4 (Certara, Princeton, NJ, USA).

The PD biomarkers, including *TRIB3*, *DDIT3* and *MAP1LC3B*, were determined in whole blood samples. Briefly, blood samples were taken at four different times: on the first day of the run-in period (day 1) before ABTL0812 intake and 8 h later, on the first day of the first (day 7) and at the second cycle of chemotherapy (day 28) before drug intake. Total RNA was isolated from whole blood samples, converted into cDNA and gene expression was assessed by quantitative PCR (qPCR). Relative mRNA expression levels were calculated using the 2^−ΔΔCt^ method and are presented as ratios to the housekeeping gene *GAPDH*. Values represented in the graph correspond to the mean of 2^−ΔΔCt^ values and its associated SEMs. Statistical analysis was performed using ΔΔCt values. The TaqMan probes used were: *GAPDH* Hs99999905_m1; *TRIB3* Hs01082394_m1, *DDIT3* Hs99999172_m1 and *MAP1LC3B* Hs00917682_m1. Statistical analyses of qPCR data were analyzed by t-test using the ΔΔCt values.

P53 status and MMR-D assessment were performed by IHC following local guidelines. Data about these biomarkers were collected retrospectively from medical records.

### Sample size calculation

For the phase 1, it was estimated that the sample size would be between 15 and 36 patients, depending on the number of de-escalation levels. A final number of 16 patients with EC and 5 patients with sq-NSCLC were recruited in 6 sites between France and Spain.

For the phase 2, the sample size calculation was based on a two-stage optimal Simon’s design with futility boundary at interim analysis. The Simon’s design was planned to attain an 80% power at 5% one-sided nominal alpha level. It was hypothesized that excluding an ORR ≤ 52% while targeting an improvement of the ORR to ≥ 72% would be an optimal approach to the evaluation of the study strategy. At least 13 evaluable patients would be accrued in the first stage. If there were less than 8 responders in these 13 patients, the study might be stopped, otherwise, 30 additional patients would be accrued. Finally, accrual was stopped at 38 evaluable patients for slow recruitment rate. Patients with EC who participated in the phase 1 were also included in the evaluation of the phase 2.

### Ethical issues

The study was initially registered on 19-Sep-2016 and approved in Spain by the National Competent Authority (AEMPS: Agencia Española de Medicamentos y Productos Sanitarios) and by the Ethics Committee “Comité de Ética de la Investigación con medicamentos (CEIm)” from Hospital Universitari Vall d’Hebrón (Barcelona, Spain), and in France by the National Competent Authority (ANSM: Agence nationale de sécurité du médicament et des produits de santé) and by the Ethics Committee “Comité de Protection des Personnes SUD-EST II” from Groupemet Hospitalier Est (Bron, France). All patients signed an informed consent before enrollment.

## Results

### Phase 1 and RP2D

Between November 2016 and March 2018, 16 patients with EC and 5 patients with sq-NSCLC were recruited in 6 sites. The median age was 68 years in both types of tumors; all sq-NSCLC patients were male. ECOG was 0 in 8 patients and 1 in 13. All patients were Caucasian and 50% were recurrent. Regarding patients with EC, 13 patients (81%) had endometrioid histology and 2 patients (12.5%) were serous (See Table 1).

**Table 1:** Patients demography

Variable		Phase I			Phase II
		Endometrial cancer	Squamous NSCLC	Overall	Endometrial cancer
	N	16	5	21	51
Age, years	Median (range)	68 (49–81)	68 (58–75)	65 (49–81)	69 (48–82)
Weight, kg	Median (range)	67.8 (46.0–103.6)	75.0 (61.0–111.3)	68.0 (46.0–111.3)	68.0 (46.0–130.0)
Height, cm	Median (range)	156 (147–163)	166 (164–175)	159 (147–175)	159 (147–170)
Gender	M/F	0/16	5/0	5/16	0/51
ECOG	0/1	6/10	2/3	8/13	23/28
Race	Caucasian	16	5	21	***51***
Ethnicity	Hispanic or Latino	2	0	2	2
	Not Hispanic or Latino	13	5	18	29
	Not reported			1	20
Country	Spain	16	5	21	31
	France	0	0	0	20
Smoking status	Never/Smoker/Ex/NR	11/2/2/1	0/1/4/0	11/3/6/1	34/4/10/3
Stage	IIIa	1 (6.3%)	0 (0.0%)	1 (4.8%)	1 (2.0%)
	IIIb	0	2 (40.0%)	2 (9.5%)	0
	IVa	0	2 (40.0%)	2 (9.5%)	0
	IVb	7 (43.8%)	2 (20.0%)	8 (38.1%)	10 (19.6%)
	Recurrent	8 (50.0%)	0 (0.0%)	8 (38.1%)	40 (78.4%)
Histology, endometrial cancer	Endometrioid	13	NA	13 (61.9%)	37 (74.0%)
	Serous	(81.3%)		2 (9.5%)	8 (16.0%)
	Clear cell	2 (12.5%)		1 (4.8%)	3 (6.0%)
	Other	1 (6.3%) ---			2 (4.0%)
Differentiation grade	1	2 (12.5%)	NR	2 (9.5%)	3 2 (5.9%)
	2	3 (18.7%)		3 (14.3%)	14 (27.4%)
	3	7 (43.7%)		7 (33.3%)	15 (29.4%)
	Unknown	4 (25.0%)		9	19 (37.2%)
Recurrent patients & Prior anticancer therapy	Recurrent	8 (50.0%)	4 (80.0%)	12 (57.1%)	40 (78.4%)
	• Chemo	3 (18.8%)	3 (60.0%)	6 (28.6%)	18 (35.3%)
	• Hormone	0	0	0	1 (2.0%)
	• Radiation	4 (25.0%)	3 (60.0%)	7 (33.3%)	26 (51.0%)
	• Surgery	8 (50.0%)	2 (40.0%)	10 (47.6%)	38 (74.5%)
	• Other	1 (6.3%)	0 (0.0%)	1 (4.8%)	2 (3.9%)

Regarding completed number of cycles, 2 patients with EC completed 8 cycles of CP, 9 patients (7 with EC and 2 with sq-NSCLC) completed 6 cycles, 2 patients with EC completed 5 cycles, 4 patients (3 with EC and 1 with sq-NSCLC) did it for 3 cycles and 4 patients (2 with EC and 2 with sq-NSCLC) for 1 cycle. The reasons for leaving the study were progressive disease (PD) in 12 patients (11 EC and 1 sq-NSCLC), patient’s withdrawal in 6 cases (3 EC and 3 sq-NSCLC), and investigator decision in 2 patients (1 EC and 1 sq-NSCLC). From those, 65% of the patients had dose intensities ≥ 75%, 24% in the range 60–75% and 1 patient had dose intensity < 60% due to dose reductions as a consequence of AEs.

**Fig. 1:**
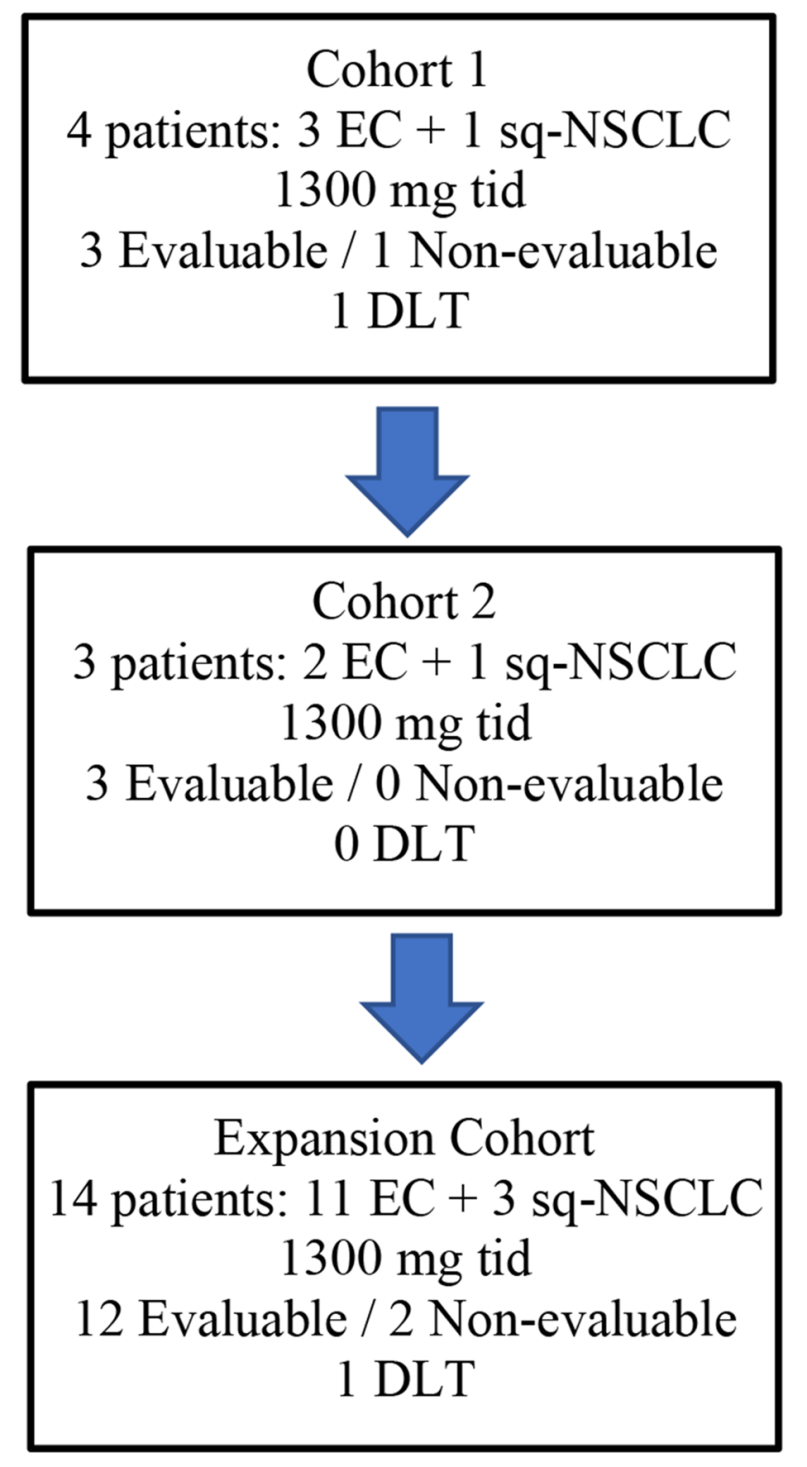
Flow chart of included patients in the Phase I part of the study. EC, endometrial cancer; sq-NSCLC, squamous-non small cell lung carcinoma; DLT, dose limiting toxicity

Initially, 4 patients were recruited in the first cohort at 1300 mg tid. One DLT (grade 4 neutropenia^2^) was observed in one patient with EC, therefore three additional patients were recruited in a second cohort at the same dose level. In this second cohort no DLTs were observed. In the expansion cohort 14 patients were recruited. One DLT (grade 3 neutropenia in a sq-NSCLC patient) was observed. Therefore, the safety endpoint was achieved and the RP2D selected was 1300 mg tid (Fig. 1). Overall safety is described in a section below.

### Safety

The combination of ABLT0812 plus CP caused at least one AE in all 51 patients and 72.5% had AEs grades 3 or 4. However, it is important to highlight that these AEs could be caused by CP. As a matter of fact, the use of ABTL0812 did not significantly increase the number of AEs during the induction phase. Regarding hematological toxicity, neutropenia was the most frequent AE and it was observed in 53% of the patients (grade 3 or 4 in 47% of all the patients). Anemia was reported in 37% of the patients (6% grade 3 or 4); thrombocytopenia was reported in 20% of the patients and (4% grade 3 or 4). Among the non-hematological AEs, nausea and asthenia were both reported in 67% of the patients (grade 3 or 4 in 4% and 2% of the patients, respectively). Diarrhea and vomiting were both reported in 55% of the patients (grade 3 or 4 in 4% and 2% of the patients, respectively) (Tables 2 and 3).

**Table 2:** Summary of adverse events

	Patients (*n* = 51)
Patients with SAEs	13 (25.5%)
Patients with AEs	51 (100.0%)
Patients with CTCAE grade:	
1 or 2 AEs	50 (98.0%)
3 AEs	34 (66.7%)
4 AEs	9 (17.6%)
Patients with AEs:	
related to study treatment not related to study treatment	50 (98.0%) 45 (88.2%)
Patients with AEs leading to drug discontinuation	6 (11.8%)
Patients with AEs leading to death	0 (0.0%)

**Table 3:** Summary of adverse events by grade that appeared in > 10% of the patients

	Any grade	Grade 1	Grade 2	Grade 3	Grade 4
Any adverse event	51 (100.0%)	3 (5.9%)	11 (21.6%)	28 (54.9%)	9 (17.6%)
**Hematological**					
Neutropenia	27 (52.9%)	0 (0.0%)	3 (5.9%)	16 (31.4%)	8 (15.7%)
Anemia	19 (37.3%)	7 (13.7%)	9 (17.6%)	3 (5.9%)	0 (0.0%)
Thrombocytopenia	10 (19.6%)	4 (7.8%)	4 (7.8%)	2 (3.9%)	0 (0.0%)
Lymphopenia	4 (7.8%)	1 (2.0%)	2 (3.9%)	1 (2.0%)	0 (0.0%)
Leukopenia	1 (2.0%)	0 (0.0%)	1 (2.0%)	0 (0.0%)	0 (0.0%)
**Non-hematological**					
Asthenia	34 (66.7%)	15 (29.4%)	18 (35.3%)	1 (2.0%)	0 (0.0%)
Nausea	34 (66.7%)	13 (25.5%)	19 (37.7%)	2 (3.9%)	0 (0.0%)
Diarrhea	28 (54.9%)	21 (41.2%)	5 (9.8%)	2 (3.9%)	0 (0.0%)
Vomiting	28 (54.9%)	17 (33.3%)	10 (19.6%)	1 (2.0%)	0 (0.0%)
Alopecia	22 (43.1%)	5 (9.8%)	17 (33.3%)	0 (0.0%)	0 (0.0%)
Arthralgia	18 (35.3%)	13 (25.5%)	5 (9.8%)	0 (0.0%)	0 (0.0%)
Neurotoxicity	15 (29.4%)	6 (11.8%)	8 (15.7%)	1 (2.0%)	0 (0.0%)
Constipation	14 (27.5%)	10 (19.6%)	4 (7.8%)	0 (0.0%)	0 (0.0%)
Stomatitis	13 (25.5%)	10 (19.6%)	3 (5.9%)	0 (0.0%)	0 (0.0%)
Dysgeusia	12 (23.5%)	8 (15.7%)	2 (3.9%)	1 (2.0%)	0 (0.0%)
Neuropathy peripheral	12 (23.5%)	7 (13.7%)	3 (5.9%)	2 (3.9%)	0 (0.0%)
Decreased appetite	11 (21.6%)	8 (15.7%)	1 (2.0%)	2 (3.9%)	0 (0.0%)
Musculoskeletal pain	9 (17.6%)	7 (13.7%)	2 (3.9%)	0 (0.0%)	0 (0.0%)
Dyspepsia	9 (17.6%)	6 (11.8%)	3 (5.9%)	0 (0.0%)	0 (0.0%)
Abdominal pain upper	8 (15.7%)	7 (13.7%)	1 (2.0%)	0 (0.0%)	0 (0.0%)
Dizziness	8 (15.7%)	7 (13.7%)	1 (2.0%)	0 (0.0%)	0 (0.0%)
Oedema	8 (15.7%)	7 (13.7%)	1 (2.0%)	0 (0.0%)	0 (0.0%)
Rash	7 (13.7%)	5 (9.8%)	2 (3.9%)	0 (0.0%)	0 (0.0%)
Insomnia	7 (13.7%)	5 (9.8%)	1 (2.0%)	0 (0.0%)	0 (0.0%)
Fatigue	7 (13.7%)	2 (3.9%)	5 (9.8%)	0 (0.0%)	0 (0.0%)
Cough	6 (11.8%)	4 (7.8%)	2 (3.9%)	0 (0.0%)	0 (0.0%)

Data indicate number and percentage of patients who experienced any type of adverse event

### Efficacy

Ten patients did not achieve the primary efficacy endpoint and very low dose intensity was observed in 3 patients; therefore, these 13 patients were not included in the per-protocol efficacy analysis group, which had a population of 38 patients. The evaluation of the first 16 patients from phase 1 safety population showed that there were 11 responses (3 CR and 8 PR), above the boundary of futility. Therefore, it was concluded that the study should not be stopped, and recruitment continued to phase 2 until all patients were available for the efficacy analysis.

**Fig. 2:**
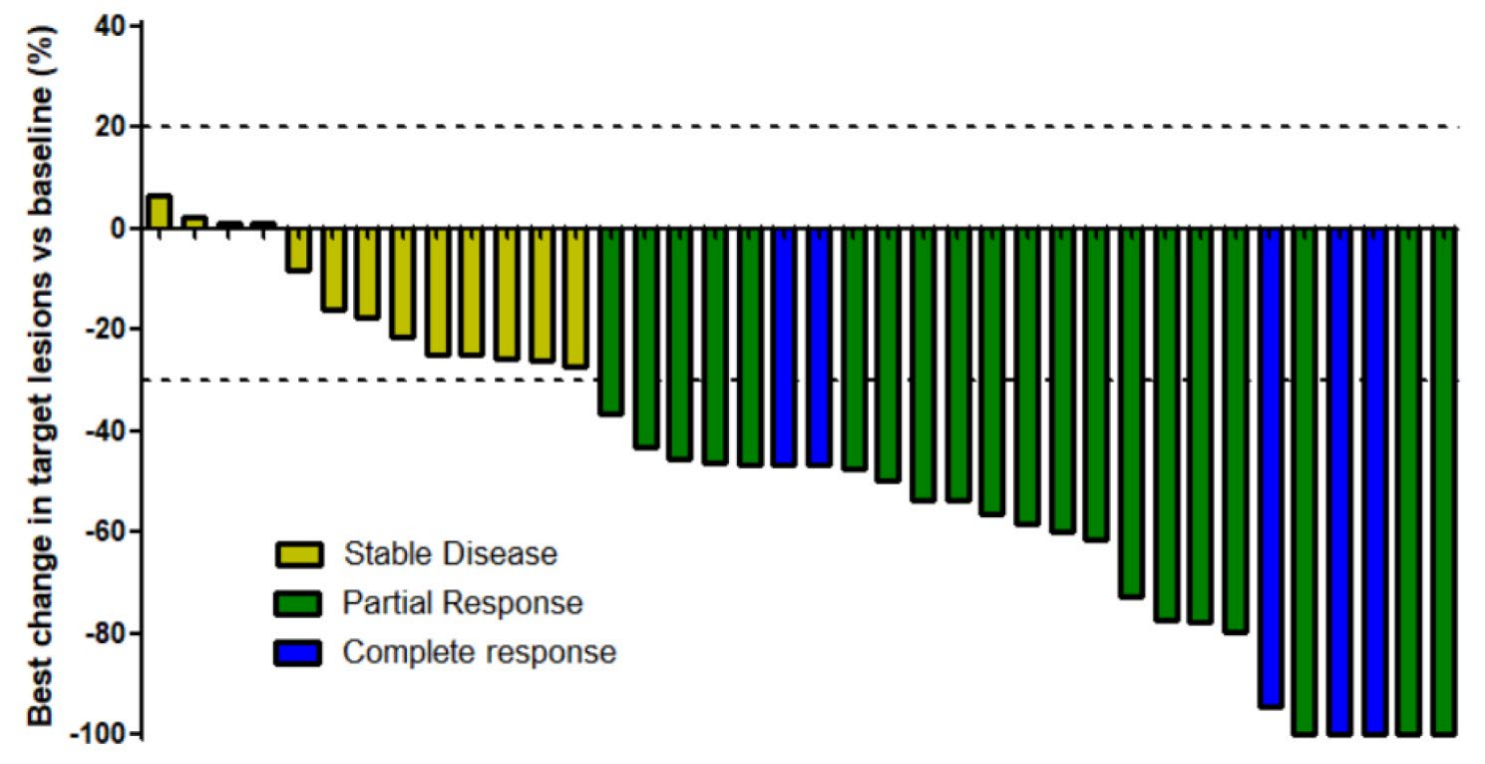
Waterfall representation of best change from baseline of target lesions in endometrial cancer patients

**Fig. 3:**
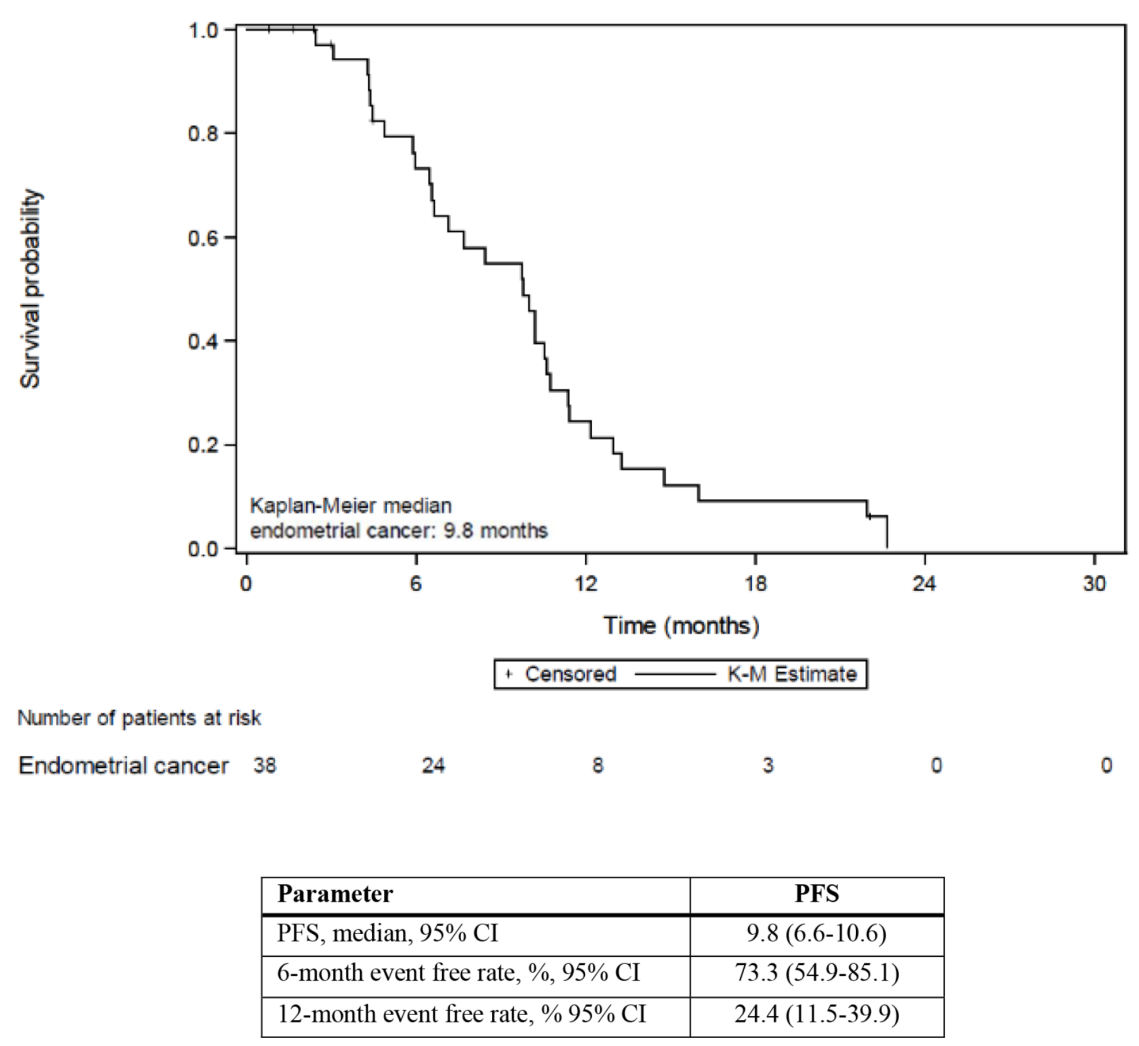
Kaplan-Meir estimate of progression free survival in endometrial cancer patients

**Fig. 4:**
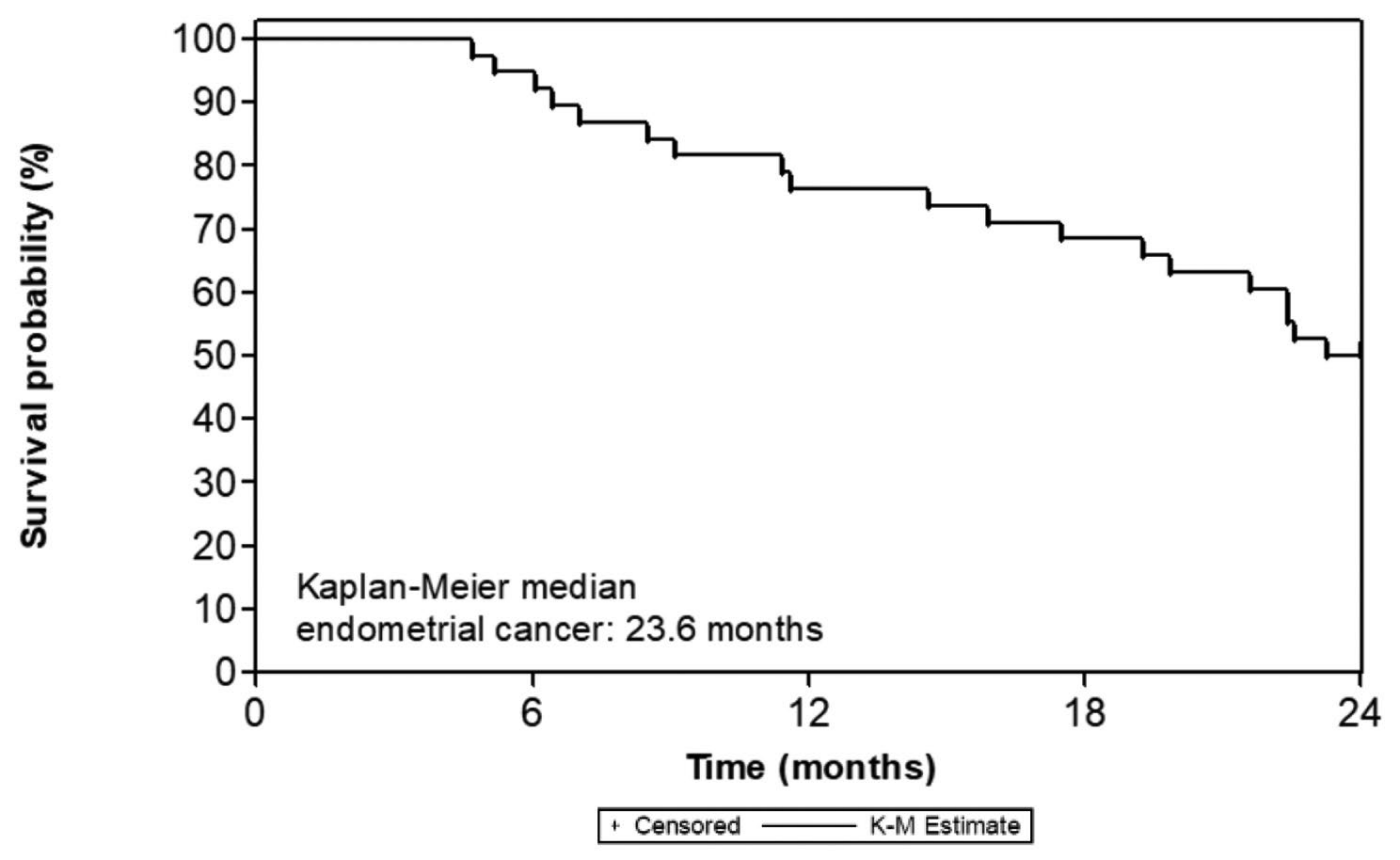
Kaplan-Meir estimate of overall survival in endometrial cancer patients

At the time of the final analysis, the observed best responses was complete response (CR) in 5 patients (13.2%), partial response (PR) in 20 patients (52.6%), and stable disease (SD) in 13 patients (34.2%); none of the patients had PD; ORR (CR plus PR) was therefore observed in 25 patients out of 38 (65.8%, 95% CI: 52.0-78.9); disease control rate (CR plus PR plus SD) was recorded in all 38 patients (100.0%) (Table 4). A waterfall plot of the patients is shown in Fig. 2. Median duration of response was 7.4 months (95% CI: 6.3–10.8 months), median PFS was 9.8 months (95% CI: 6.6–10.6 months); event free rate was of 73.3% at 6 months and 24.4% at 1 year; no progression event was reported in 6 patients who left the trial for reasons other than disease progression and were censored (Table 2; Figs. 2, 3 and 4). Median OS was 23.2 months (95% CI 6.4-ND); event free rate was 74.9% at 1 year. Additional subpopulation analysis of p53 wildtype vs. mutated patients and MMR proficient vs. deficient patients did not find any significant difference between subpopulations (data not shown).

**Table 4:** Response rate summary

Parameter	Response *n* = 38
Complete Response, n (%)	5 (13.2%)
Partial Response, n (%)	20 (52.6%)
Stable Disease, n (%)	13 (34.2%)
Progressive Disease, n (%)	0 (0%)
Overall Response Rate, n (%)	25 (65.8%)
95% CI	52.0-78.9
Disease Control Rate, n (%)	38 (100.0%)
95% CI	90.8–100.0
Duration of Response, median	7.4 months
95% CI	6.3–10.8 months

CI: Confidence interval

### Pharmacokinetic and pharmacodynamic biomarkers

**Fig. 5:**
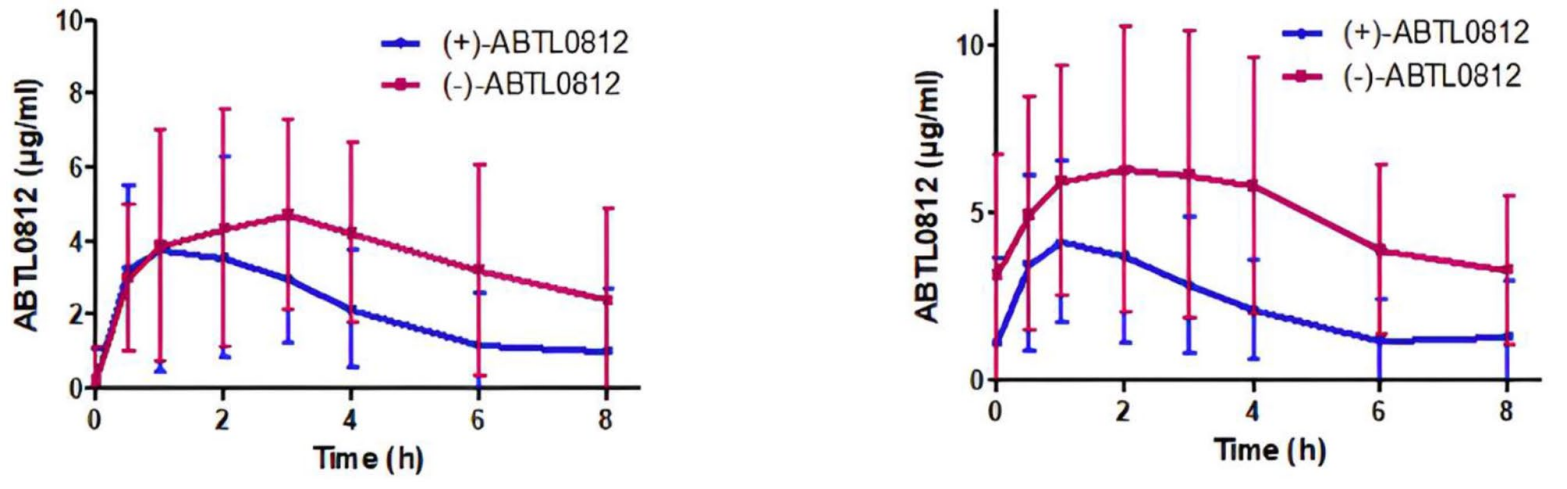
ABTL0812 plasma levels of its (+) and (-)-enantiomers after single administration (left panel) and 28-day administration 1300 mg tid

For PK analysis, ABTL0812 concentration was measured in blood samples as a single dose and during chronic treatment tid. Blood samples were taken from patients after the first ABTL0812 administration (single) and at approximately 28 days of drug administration at different timepoints from 0 to 8 h. ABTL0812 C_max_ and C_min_ plasma levels were in the micromolar range, as well as AUC. No significant differences were observed in the pharmacokinetic parameters after single and chronic administration suggestive of drug accumulation (Table 5; Fig. 5).

**Table 5:** Pharmacokinetic parameters of ABTL0812 enantiomers after single and chronic administration

Parameter	Single Dose		Chronic administration	
	(-)-ABTL0812	(+)-ABTL0812	(-)-ABTL0812	(+)-ABTL0812
AUC (µg·h/ml)	32.6 ± 13.6	17.6 ± 7.0	56.0 ± 36.4	16.3 ± 8.1
C_max_ (µg/ml)	7.0 ± 3.0	5.5 ± 2.9	8.0 ± 4.5	4.9 ± 2.7
C_min_ (µg/ml)	2.2 ± 2.4	0.9 ± 1.6	3.3 ± 2.2	1.3 ± 1.7
T_1/2_ (h)	2.0 ± 0.7	1.4 ± 0.8	3.3 ± 1.8	2.1 ± 1.3
T_max_ (h)	3.2 ± 2.1	2.4 ± 2.0	3.3 ± 2.3	2.0 ± 1.6

**Fig. 6:**
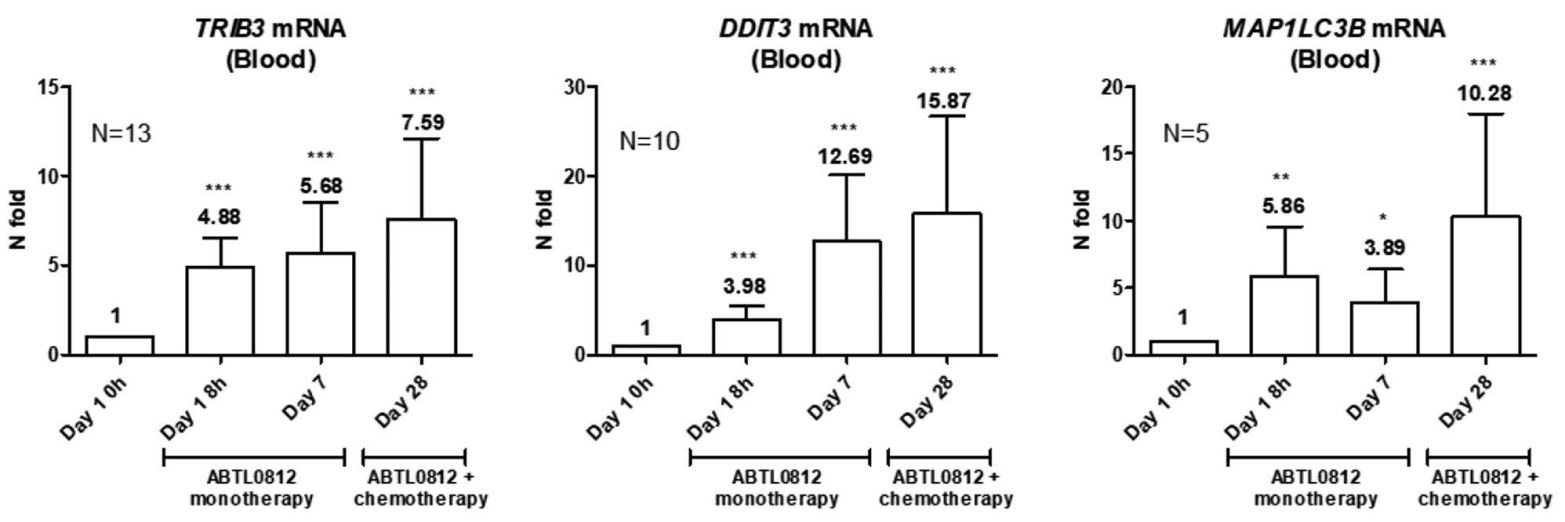
ABTL0812 pharmacodynamic biomarkers analyzed in blood samples from endometrial cancer patients. *TRIB3*, *DDIT3* and *MAP1LC3B* mRNA expression levels were evaluated by quantitative PCR in mRNA from whole blood samples. Values represented in the graph correspond to the mean of 2^−ΔΔCt^ values and its associated SEMs. Statistical analysis was performed using ΔΔCt values. * *p* ≤ 0,05; ** *p* ≤ 0,01; *** *p* ≤ 0,001 vs. baseline levels (Day 1 0 h) by t-test

Based on the mechanism of action of ABTL0812 and the preclinical data, several PD biomarkers candidate were investigated in blood samples as a surrogate tissue. In this context, the mRNA levels of the UPR markers*TRIB3* and *DDIT3* (previously described to be upregulated by ABTL0812 [12]), as well as the autophagy marker *MAP1LC3B*, were analyzed in patients’ whole blood samples. The expression of the three genes was induced by ABTL0812 after 8 h, and this upregulation was sustained after 7 days of single therapy, and at day 28 when ABTL0812 had been already administered in combination with chemotherapy for 21 days. This sustained and robust induction of these surrogate biomarkers support the pharmacological action of ABTL0812, and validates *TRIB3*, *DDIT3* and *MAP1LC3B* as PD biomarkers to monitor ABTL0812 treatment in humans (Fig. 6).

## Discussion

In the present study the safety and preliminary efficacy of the autophagy inducer ABTL0812 in combination with CP in patients with advanced and recurrent EC was evaluated.

ABTL0812 novel mechanism of action offers an attractive therapeutic approach due to the high frequency of alterations in the PI3K/AKT pathway found in EC [16, 17]. In this context, a maintenance strategy targeting the PAM pathway with ABTL0812 could be of interest in EC as supported by the previous preclinical data [13]. Moreover, in the dose-finding phase 1 trial with ABTL0812 monotherapy, the FIH study, an EC patient harboring mutations in *PTEN* (Met264Ile), *PIK3CA* (Arg88Gln) and *Akt1* (Glu17Lys) obtained an important clinical benefit from ABTL0812 500 mg qd with a disease stabilization for 60 weeks [14], suggesting a potential predictive role of PAM alterations.

 The chemotherapy combination CP is one of the main backbones used in the first line therapy of patients with recurrent/metastatic EC [1]. ABTL0812 has shown additive or synergistic therapeutic effect in different preclinical models of endometrial cancer with both agents. Very importantly, when those models were performed in vivo, no evidence of potentiation of toxic effects were observed [13].

In this context it is considered justified to test the combination of ABTL0812 plus CP in EC. The de-escalation, and not a conventional escalation, phase 1 design was decided according to the favorable safety profile at high dose and the previous data from the phase 1 (FIH) in which the RPD2 of the drug as single agent was established at 1300 mg tid. At this dose level no DLTs occurred, and only 1 out of 29 patients had grade 3 related-adverse events (increase of hepatic enzymes), while all other drug-related were grade 1 and 2. Finally the de-escalation design avoided a potential confounding effect of the chemotherapy combination of CP in the MTD that could lead to an infra-therapeutic RP2D of ABTL0812.

The toxic profile of the combination ABTL0812 plus CP in this study was acceptable when ABTL0812 was dosed at 1300 mg tid. Only one DLT was reported in the de-escalation part, a grade 4 neutropenia. This AE would not have been reported as DLT after the protocol was amended, since its duration was inferior to 7 days. Since only one additional DLT was reported in the 12 evaluable patients of the expansion part, the study proceeded to the phase 2. An important limitation is that it is complex to identify which drug could be related with every AE. The combination of CP is related with a high proportion of hematological AEs. In this context it is uncertain whether ABTL0812 increases the toxic profile of CP. In fact, the patient that experienced a grade 4 neutropenia continued presenting different grades of neutropenia after withdrawal of ABTL0812.

Noteworthy to mention, one important issue is that the formulation of ABTL0812 in this trial was in a liquid solution. This formulation caused a discomfort and upper gastrointestinal AEs (mainly grade 1–2) that impacted in the adherence and lead to discontinuation of the trial in 10 patients. The development of a new oral ABTL0812 formulation in capsules started when this study was very advanced. This new solid formulation is currently being evaluated in a new clinical trial (NCT04431258), and preliminary results indicate that the frequency of these AEs is reduced.

The treatment of patients with ABTL0812 and chemotherapy continued until disease progression, unacceptable toxicity or IC withdrawal. All 51 patients had at least one AE of any grade, 14 patients (27.5%) had grade 1–2 AEs and 38 patients (72.5%) grade 3–4 AEs, while no grade 5 AEs were reported. The hematological AEs that showed the highest incidence were neutropenia of any grade, observed in 27 patients (52.9%), anemia in 19 patients (37.3%) and thrombocytopenia in 10 patients (19.6%). Interestingly, this incidence was markedly lower than that reported for the same hematological AEs in the GOG0209 trial where neutropenia, anemia and thrombocytopenia incidences were 93.1%, 93.2% and 62.8% of the patients, respectively. Regarding non-hematological AEs, the gastrointestinal system was the most affected (after asthenia, 66.7%) as nausea in 34 patients (66.7%), diarrhea in 28 patients (54.9%) and vomiting in 28 patients (54.9%) appeared in more than half of the patients. On the contrary these gastrointestinal AEs had lower incidence in the GOG0209 study with incidences of 59.9%, 28.3% and 30.9%, respectively [3]. Altogether this analysis suggests that no potentiation of hematological AEs appears when ABTL0812 is combined with paclitaxel/carboplatin, while a tendency to a higher frequency of gastrointestinal AEs was observed with the combination ABTL0812 plus paclitaxel/carboplatin. Nonetheless, these findings should be approached with caution due to various factors, such as spatial-temporal disparities and differences in population.

All patients included in this study were required to have measurable disease and tumor response was analyzed by investigator analysis following RECIST v1.1 criteria. An ORR of 65.8% was observed, with a DCR of 100% at 16 weeks and median DOR was 7.4 months. Survival parameters have shown that median PFS was 9.8 months and median OS 23.2 months. To date, the larger phase 2 study evaluating carboplatin plus paclitaxel in patients with advanced/recurrent endometrial cancer is the GOG0209 study [3]. Intertrial comparisons are difficult as the timing of therapy administration and the population characteristics are not reproducible in one trial vs. other. In this context, the population of the GOG0209 study was enriched in stage III (41.7% vs. 2.0% in our study) and in newly diagnosed stage IV tumors (30.1% vs. 19.6% in our study). However, the proportion of recurrent tumors is much higher in our study (28.3% in GOG vs. 78.4% in our study). Even in this population with a potentially poorer prognosis, our results are similar to that obtained with CP chemotherapy in the GOG0209 in which ORR was 52%, PFS was 13.2 months, and OS 37.0 months. In the GOG0209 the OS was 20.4 months in those patients with measurable or recurrent disease, which is the most similar population with that of this study, while it was 113 months in patients with no measurable or not recurrent disease. Two recent phase 3 trials, the RUBY and the NRG-018 trials, compared CP +/- an immune checkpoint inhibitor (pembrolizumab or dostarlimab) in the first line setting of advanced or recurrent EC. Both studies showed a significant increase in PFS and OS with the addition of the immune checkpoint. In the MMR-D benefit was substantial. The control arms in these 2 Phase 2 trials evaluating in patients with advanced/metastatic EC and measurable disease reported a median PFS of 8–9 months [18, 19]. However, these trials also highlight how quickly the treatment landscape is evolving in EC. In our retrospective analysis ABTL0812 was active in both MMR-D and MMR-P patients, although given the small numbers these data should be considered with caution. When compared the efficacy of ABTL0812 plus CP with the placebo arm of both trials, an increase of PFS is observed (9.8 months vs. 8.5 months in NGR-018 trial or ~ 7.8 months in RUBY trial), specially for the pMMR population [18, 19]. Moreover, an increase in ORR (65.8% in this study vs. 64.8% in RUBY trial) and DCR (100% in this trial vs. 87.6% in RUBY trial) was also true when compared both assays, especially in the pMMR population. Overall, the difference of staging and measurable disease precludes to compare all three studies with this one, however these data may suggest that for similar populations ABTL0812 could potentially have an additive effect to CP.

The pharmacokinetic levels observed in the patients of the trial confirms the results observed in the FIH study with ABTL0812 as single agent in patients with advanced solid tumors [14], suggesting no interaction with the chemotherapy, and supporting the activity against EC cells in preclinical models [13]. Biomarkers of activity are quickly activated, since as soon as 8 h after the first administration, a significant activation of *TRIB3* and *CHOP* is observed, which is sustained at least up to 28 days after starting treatment and the patients have received two chemotherapy cycles. Altogether, the pharmacokinetic and pharmacodynamic analysis suggests that the doses administered are compatible with drug efficacy.

An important limitation in this study is the absence of prospectively included information regarding molecular classification of EC. Although data from p53 status and MMR-D were obtained, this was extracted retrospectively from medical records of analysis performed locally. It would be of interest to develop in a prospective setting the impact of ABTL0812 in every different subtype of patients with EC.

In summary, the present phase 1/2 study of ABTL0812 in patients with advanced/recurrent endometrial cancer suggests that ABTL0812 does not add significant toxicities to the standard chemotherapy for CP. Preliminary efficacy data suggest encouraging activity of this combination in EC. Therefore, the improvement of the benefit-to-safety ratio observed for of ABTL0812 plus CP warrants further clinical investigation of this combination.

## Data Availability

The datasets used and/or analyzed during the current study are available on reasonable request from the corresponding author.

## Footnotes

1 Before version 4.0 of the protocol, grade 4 neutropenia did not include the temporal threshold of 7 days.

2 The neutropenia that this patient suffered was considered as DLT in spite that it lasted less than 7 days because at that moment version 3 of the protocol was in place that did not consider neutropenia duration.

## Abbreviations

CP: carboplatin and paclitaxel
EC: endometrial cancer
sq-NSCLC: squamous non-small cell lung cancer
AUC: area under the curve
DOR: duration of response
PFS: progression-free survival
OS: overall survival
DLT: dose limiting toxicity
RP2D: recommended phase 2 dose
AE: adverse events
ORR: overall response rate
PK: pharmacokinetics
PD: pharmacodynamic
FiH: first in humans
MTD: maximum tolerated dose (MTD)
ULN: upper limit of normal
CR: complete response
PR: partial response
SD: stable disease
