# Correction: ENDOLUNG trial. A phase 1/2 study of the Akt/mTOR inhibitor and autophagy inducer Ibrilatazar (ABTL0812) in combination with paclitaxel/carboplatin in patients with advanced/recurrent endometrial cancer: Alexandra Leary

**DOI:** 10.1186/s12885-024-12700-0

**Published:** 2024-07-29

**Authors:** Alexandra Leary, Purificación Estévez-García, Renaud Sabatier, Isabelle Ray-Coquard, Margarita Romeo, Pilar Barretina-Ginesta, Marta Gil-Martin, Elena Garralda, Joaquim Bosch-Barrera, Teresa Morán, Paloma Martin-Martorell, Ernest Nadal, Pere Gascón, Jordi Rodon, Jose M Lizcano, Pau Muñoz-Guardiola, Gemma Fierro-Durán, Oriol Pedrós-Gámez, Héctor Pérez-Montoyo, Marc Yeste-Velasco, Marc Cortal, Antonio Pérez-Campos, Jose Alfon, Carles Domenech, Alejandro Pérez-Fidalgo, Ana Oaknin

**Affiliations:** 1 Medical Oncology, Institut Gustave Roussy, Villejuif, France; 2 Medical Oncology, Hospital Universitario Virgen del Rocío, Sevilla, Spain; 3 Department of Medical Oncology, Aix-Marseille Univ, Inserm, CNRS, Institut Paoli-Calmettes, 232 Boulevard Sainte Marguerite, Marseille, France; 4 Medical Oncology, Centre Léon Bérard, Lyon, France; 5 Department of Medical Oncology, Department of Medicine, Institut Català d’Oncologia Badalona Hospital Germans Trias i Pujol, Badalona-Applied Research Group in Oncology, Germans Trias i Pujol Institute, Universitat Autònoma de Barcelona, Badalona, Spain; 6 Medical Oncology Department, Precision Oncology Group (OncoGIR-Pro) Girona Biomedical Research Institute (IDIBGI) and Department of Medical Sciences, Institut Català d’Oncologia (ICO), Medical School University of Girona (UdG), Girona, Spain; 7 Medical Oncology, Catalan Institute of Oncology and IDIBELL, L’Hospitalet del Llobregat, Barcelona, Spain; 8 Medical Oncology Service, Vall d’Hebron Institute of Oncology (VHIO), Vall d’Hebron Barcelona Hospital Campus, Barcelona, Spain; 9 Medical Oncology, INCLIVA-Hospital Clínico Universitario, Valencia, Spain; 10 Medical Oncology Service, Hospital Clínic. Universitat de Barcelona, Barcelona, Spain; 11 Early Drug Development, The University of Texas M. D. Anderson Cancer Center, Houston, TX USA; 12 Department of Biochemistry and Molecular Biology, Institut de Neurocièncias, Universitat Autònoma de Barcelona, Cerdanyola del Vallès, Barcelona, Spain; 13 Protein Kinases in Cancer Research, Vall Hebron Institut de Recerca (VHIR), Barcelona, Spain; 14 Research and Development, Ability Pharmaceuticals, Cerdanyola del Vallès, Barcelona, Spain

**BMC Cancer (2024) 24:876**

10.1186/s12885-024-12501-5

Following publication of the original article [1], the authors reported the the equal contribution statement was missing in the original submission. The correct equal contribution statement is:

Alejandro Pérez-Fidalgo and Ana Oaknin contributed equally to this work.
